# Transcriptomic profiling of neural cultures from the KYOU iPSC line via alternative differentiation protocols

**DOI:** 10.3389/fnmol.2025.1661986

**Published:** 2025-10-28

**Authors:** Adelya Galiakberova, Sergey Ivanov, Arkadiy Golov, Alexander Artyuhov, Alexey Zolkin, Nikolay Kondratyev, Alexey Lagunin, Vera Golimbet, Erdem Dashinimaev

**Affiliations:** ^1^Pirogov Russian National Research Medical University, Moscow, Russia; ^2^Institute of Biomedical Chemistry, Moscow, Russia; ^3^Mental Health Research Center, Moscow, Russia; ^4^Research Institute of Molecular and Cellular Medicine, RUDN University, Moscow, Russia; ^5^Moscow Center for Advanced Studies, Moscow, Russia; ^6^Banzarov Buryat State University, Ulan-Ude, Russia

**Keywords:** iPSC, neural differentiation, DUAL SMAD inhibition, *NGN2*, RNA-seq

## Abstract

The differentiation of pluripotent stem cells into neurons is an essential area of biomedical research, with significant implications for understanding neural development and treating neurological diseases. This study compares neural cultures derived from a common induced pluripotent stem cell line (KYOU-DXR0109B) generated by two widely adopted methods: DUAL SMAD inhibition and exogenous *NGN2* overexpression. The DUAL SMAD inhibition method, which differentiates through the neural stem cell stage, produces heterogeneous cultures containing a mix of neurons, neural precursors, and glial cells. Conversely, *NGN2* overexpression generates more homogeneous cultures composed predominantly of mature neurons. Transcriptomic analysis revealed significant differences in neural gene markers expression profiles, with cultures from the DUAL SMAD inhibition method enriched in neural stem cell and glial markers, while *NGN2* overexpression cultures showed elevated markers for cholinergic and peripheral sensory neurons. This study underscores the importance of choosing appropriate differentiation protocols based on the desired cell types, as each method yields neural cultures with distinct cellular compositions. Understanding these differences can help optimize protocols for specific research and therapeutic applications.

## 1 Introduction

There is a wide variety of different methods and protocols for differentiation of pluripotent stem cells (PSCs) into neurons *in vitro* (Chambers et al., 2009; Zhang et al., 2013; Galiakberova and Dashinimaev, 2020). Globally, two main approaches to 2D generation of neurons from PSCs can be distinguished. The first one is differentiation of PSCs into neurons through the neural stem cell (NSC) stage, and the second one is direct differentiation of PSCs into neurons.

One of the most popular methods of the first approach is the DUAL SMAD inhibition method developed by Chambers and colleagues in 2009 (Chambers et al., 2009). This method is based on inhibition of two pathways of SMAD signaling activation (Lefty/Activin/TGFβ- and BMP-signaling pathways) in PSCs using small molecules (Smith et al., 2008; Yu et al., 2008; Chambers et al., 2009). This inhibition results in the differentiation of PSCs into neuroectoderm cells and their derivatives - neural stem cells. The obtained NSCs can be cultured for some number of passages or directed to terminal differentiation. This approach in a sense mimics the step-by-step processes of neurogenesis. As a result, neural cultures can be obtained, in which, however, contain not only neurons, but also various types of neural precursors as well as glial cells (Chambers et al., 2009; Sudina et al., 2024). In addition, despite the simplicity of this approach, it is quite time-consuming.

The approach with direct differentiation of PSCs into neurons involves exogenous overexpression of transcription factors that determine neuronal fate, such as Neurogenin 2 (NGN2) (Zhang et al., 2013; Lin et al., 2021). Protocols for exogenous overexpression of the *NGN2* gene can use plasmids, viruses, synthetic mRNA or CRISPR genome editing technologies to obtain constitutive, inducible or temporary expression. The best known and used protocol is lentiviral transduction of PSCs with the TetON system for tetracycline-regulated transgene expression (Urlinger et al., 2000; Zhang et al., 2013). Induction of temporary overexpression of *NGN2* in PSCs leads to their rapid differentiation into neurons without delaying the cells at the precursor stage. At the same time, the resulting neural cultures are mainly composed of neurons and do not contain glial cells (Zhang et al., 2013). Despite the rapidity and efficiency, this method is labor-intensive due to the need to obtain transgenic PSCs expressing *NGN2* as part of the TetON system.

Although both of the aforementioned neuron generation approaches are quite actively used and studied, researchers do not always have an understanding of which approach should be used for specific tasks. In addition, each approach has many different protocols and modifications, which may affect the final outcome of these approaches. In this study, we compared the transcriptomic profiles of neural cultures derived from the same induced pluripotent stem cell (iPSC) line using two different approaches: an approach with differentiation of neurons through the stage of NSCs derived from iPSCs by DUAL SMAD inhibition method (N-DSi), and an approach with exogenous *NGN2* overexpression based on lentiviral transgene delivery within a tetracycline-activated system (iN-NGN2).

## 2 Materials and methods

### 2.1 iPSC culture

The iPS-KYOU cell line was obtained from the ATCC cell bank. Initially iPS-KYOU was generated in the Shinya Yamanaka laboratory (Kyoto University, Japan) using retroviral transfection of adult female skin fibroblasts. (KYOU-DXR0109B, ACS-1023™, ATCC®, United States).

IPS cells were cultured under standard conditions (at 37 °C in a CO2-incubator with 5% CO2). For passaging cells were incubated with Accutase™ сell detachment solution for 5 min at 37 °C, then washed twice in DPBS (PanEco) solution with centrifugation. The precipitate was resuspended in growth medium, counted and plated on dishes coated with matrigel solution in mTeSR™1 medium (Stem Cell Technologies). The medium was changed every day. Rock-inhibitor Y-27632 (5 μM; Abcam) was supplemented to the medium on the day of passaging and the next day.

### 2.2 Generation NGN2-induced neurons

#### 2.2.1 Lentivirus preparation and lentiviral transduction

The IPSC line containing a transgenic NGN2 cassette under the TetON tetracycline-inducible promoter was generated using rtTA-N144 and TRET-hNgn2-UBC-PuRo plasmids obtained from the Addgene depository. The rtTA-N144 plasmid was a gift from Andrew Yu (Addgene plasmid #66810; http://n2t.net/addgene:66810; RRID: Addgene_66,810). The pLV_TRET_hNgn2_UBC_Puro plasmid was a gift from Ron Weiss (Addgene plasmid #61474; http://n2t.net/addgene:61474; RRID: Addgene_61,474). To assemble lentiviral particles, we used HEK293TN packing cells [kindly provided by Dr. A. P. Bolshakov (INHA RAS, Moscow, Russia)]. On day 0, cells were seeded in an amount of 10^6 cells in a 6-cm Petri dish. On day 1, cells were co-transfected with three helper plasmids [pLP1 — 4 μg, pLP2 — 2 μg, pVSVG — 1 μg (Invitrogen)] and 4 μg of lentiviral vector DNA by mixing the plasmids in 300 μL of serum-free OPTI-MEM medium (Gibco) using Lipofectamine2000 reagent (Invitrogen), based on a ratio of 11 μL of reagent per 11 μg of total plasmid mixture. The plasmid and reagent mixture was then added to cells in complete growth medium [DMEM (PanEco), 10% FBS (Capricorn), GlutaMax (1 mM; Gibco), sodium pyruvate (1 mM; Gibco), PenStrep (50 μg/mL; Gibco)]. Transfection was performed for 4 h, after which the cell medium was changed to fresh complete growth medium. After 72 h (Day 4), the culture medium containing packed lentiviruses was collected, centrifuged at 100 g for 5 min, and sterilised through a 0.45 μm filter.

#### 2.2.2 Generation of TetON-NGN2 expressing iPSCs

For lentiviral transduction of iPSC, growth medium (mTeSR™1) was changed to a mixture (1:1) of mTeSRTM1 with lentivirus-containing supernatant and supplemented with Polybrene (5 μg/mL). After 24 h the medium was changed to mTeSR™1 supplemented with 5 μM Rock-inhibitor. iPS cells expressing TetON-*NGN2* were maintained in the presence of selective antibiotics – Puromycin (0.5 μg/mL; Sigma) and Hygromycin B (50 μg/mL; Serva).

#### 2.2.3 Neural differentiation of iPS-tetON-NGN2

For neuronal differentiation of iPSC-TetON-NGN2 was performed as described in our previous work (Galiakberova et al., 2022). Briefly, cells were plated on matrigel-coated petri dishes in mTeSR™1 medium supplemented with Y-27632 (5 μM) (day 0). Doxycycline (1 μg/mL; Sigma) was added from day 0 to day 5 to induce *NGN2* transgene expression. The medium was changed every day. To inhibit proliferation of undifferentiated iPSCs, Cytosine-β-d-arabinofuranoside (Ara-C) (0.1 μg/mL; Sigma) was added to the culture medium on days 2 and 3. On day 4 differentiating cultures were dissociated with Accutase™ сell detachment solution. Detached and washed cells were plated into new petri dishes, subsequently pre-coated with poly-D-Lysine (Gibco) and Matigel solutions in mixed N2B27 [Neurobasal medium (Gibco), DMEM/F12 (PanEco), GlutaMax (1 mM; Gibco), sodium pyruvate (1 mM; Gibco), PenStrep (50 μg/mL; Gibco), β-Mercaptoethanol (0.1 mM; Sigma-Aldrich), N2-supplement (100x; Gibco), B27-supplement (50x; Gibco)] and mTeSR™1 (1:1) supplemented with human BDNF (10 ng/mL; Petrotech), NGF (20 ng/mL; Petrotech), Y-27632 (5 μM) and doxycycline (2 μg/mL).

On day 5 the medium was changed to N2B27 medium supplemented with human BDNF (10 ng/mL; Petrotech), NGF (20 ng/mL; Petrotech), Y-27632 (5 μM) and doxycycline (1 μg/mL). From day 6 neuronal cultures were maintained without doxycycline. After day 7, half the culture medium was changed, twice a week.

### 2.3 Induction of neural differentiation of iPSCs using the dual SMAD inhibition method

Neuronal differentiation of iPSCs through the stage of NSCs obtained using the method of DUAL SMAD inhibition was performed as described (Galiakberova et al., 2023). In summary, for DUAL SMAD induction of iPSCs commercially available Neural Induction Medium kit (PSC Neural Induction Medium, Life Technologies) was used. Obtained NSC were cultured in Neural Proliferation Medium (NPM) [DMEM/F12 (Capricorn) with DMEM (Capricorn) v1:1, B27 Supplement (50x, Gibco), 20 ng/mL bFGF (Gibco), 20 ng/mL EGF (Gibco), Sodium Pyruvate (1 mM; Gibco), PenStrep (50 μg/mL; Gibco)] supplemented with Y-27632 (5 μM) for 5 passages. After the 5th passage, NSCs were allowed to go into spontaneous terminal differentiation. For this purpose, at day 0 of differentiation we seeded NSCs at a low density (40 × 10^3^ cells/cm^2^) on a new matrigel-coated plastic surface in neural differentiation medium N2B27 [Neurobasal (Gibco) with DMEM/F12 (Capricorn), v1:1, GlutaMax (1 mM; Gibco), Sodium Pyruvate (1 mM; Gibco), PenStrep (50 μg/mL; Gibco), β-Mercaptoethanol (0.1 mM; Sigma-Aldrich), N2-supplement (100x; Gibco), B27-supplement (50x; Gibco)] without growth factors but supplemented with Y-27632 (5 μM).

### 2.4 Total RNA extraction

Cells were detached using Accutase™ сell detachment solution (Stem Cell Technologies, Canada) washed with DPBS twice and collected by centrifugation. For total RNA extraction we used the ExtractRNA kit (Evrogen, Russia), according to the manufacturer’s instructions. The concentration of isolated RNA was measured using an Implen P360 spectrophotometer.

### 2.5 Quantitative RT-PCR analysis

We used 1 μg of total RNA for cDNA synthesis. After DNaseI (ThermoFisher, USA) treatment we performed reverse transcription with oligo-dT primers with MMLV-RT Kit (Evrogen, Russia), according to the manufacturer’s instructions. Sequences of primers for the genes of interest are listed in Table 1. Six genes were tested previously as a neuron qPCR data normalization housekeeping genes *(ACTb, C1orf43, EMC7, GAPDH, PSMB4, REEP5)* to find suitable normalization factors (Artyukhov et al., 2017; Artyuhov et al., 2019). According to geNorm software indications, *EMC7* and *PSMB4* were chosen for normalization. We used the ΔΔCt method for calculation of relative expression. First N-DSi sample was used as a calibrator. Statistical significance was calculated according to Welch’s test using SciPy.

**Table 1:** List of primer sequences used in this study.

	Gene	Sequence 5′ → 3’
1.	*ASCL1*	ATCCTAACCAGTTCGGGGAT
		TGGTGGCCTCTTGATCTCAC
2.	*MAP*	CTCAGCACCGCTAACAGAGG
		CATTGGCGCTTCGGACAAG
3.	*TUBB3*	CCGAAGCCAGCAGTGTCTAA
		AAGACAGAGACAGGAGCAGC
4.	*GFAP*	AGGTCCATGTGGAGCTTGAC
		GCCATTGCCTCATACTGCGT
5.	*NEUROD1*	ACAGATTTGCAATGGCTGGC
		CAGGTGAAATTCCCACAGCC
6.	*PAX6*	AGTGCCCGTCCATCTTTGC
		CGCTTGGTATGTTATCGTTGGT
7.	*S100B*	GAAGGGAGGGAGACAAGCAC
		TCGTGGCAGGCAGTAGTAAC
8.	*SOX1*	AAATACTGGAGACGAACGCC
		AACCCAAGTCTGGTGTCAGC
9.	*TH*	GCCCTACCAAGACCAGACGTA
		CGTGAGGCATAGCTCCTGA
10.	*BRN2*	GGGGGAAAACCCTAGACCTT
		GTCCACCTAGTTCCACTGATGT
11.	*NGN2*	GAGTTTGCAGAGCGGACTGA
		GGCATTGTGACGAATCTGGG
12.	*OCT4*	ACCCACACTGCAGCAGATCA
		CACACTCGGACCACATCCTTCT
13.	*SOX2*	TGCGAGCGCTGCACAT
		GCAGCGTGTACTTATCCTTCTTCA
14.	*MAPT*	TTTGGTGGTGGTTAGAGATATGC
		CCGAGGTGCGTGAAGAAATG
15.	*NES*	CAACAGCGACGGAGGTCTC
		GCCTCTACGCTCTCTTCTTTGA
16.	*ZIC3A*	ATGAGTAAGGCCAGTTGAGCA
		AAACCTAGAGCATTGCCCCTT
17.	*ZIC3B*	GCATGTGCATACCTCGGACA
		ACCAAGCAGGACAACACTTCA
18.	*PTN*	GGGAAGCAGAGCATGTCCTA
		ACAAATGCTTCTGCCAAAGTGA
19.	*CD44*	TCAGAGCACACCCTTCCTCT
		CCAATAAGTGCTTTCAACTCAGCA
20.	*PTPRZ*	ATACTGCCCTAGTGTCTCCATG
		AGAAAACTGGTAGAGTAAGACCAGC
21.	*SOX3*	TGGAGAACTGCAACGCCTAC
		CTCCCCACTACCCAAACGAA
22.	*PLP1*	AGGCTGCATAGAAGGAGGAGA
		TGCATGTGAGGTTTTCAGGGA
23.	*VCAM*	TTTGACAGGCTGGAGATAGACT
		TCAATGTGTAATTTAGCTCGGCA
24.	*DCX*	TGCCATGTGTGTAAGGTGCT
		GCTCTTTGGCTGCCTGGTAT
25.	*NSE*	CCGGGAACTCAGACCTGATC
		CTCTGCACCTAGTCGCATGG
26.	*PSMB4*	CATTCCGTCCACTCCCGATT
		CGAACTTAACGCCGAGGACT
27.	*EMC7*	AAAGGAGGTAGTCAGGCCGT
		GTTGCTTCACACGGTTTTCCA

### 2.6 RNA-seq

cDNA libraries were made starting with 1 μg of RNA with NEBNext Ultra II Directional RNA Library Prep (New England Biolabs, USA) and barcoded with index primers for pooled sequencing.

The gene-level counts were estimated with the “salmon” tool (version 1.2.1). During data preparation, genes with insufficient reads (not expressed in the analyzed cells) were removed using the filterByExpr function from the R edgeR package (version 3.42.0). For clustering of samples and genes, Pearson correlation coefficient values as a similarity measure and Ward’s method were used. The heatmap.2 function from the gplots package (version 3.1.3) was used to construct a heatmap of the clustering. To perform principal component and multidimensional scaling analysis, and create heatmaps, read counts values were transformed to log2-counts per million (logCPM) using voom function from the R limma package (version 3.54.2). Differentially expressed genes were identified using the approach implemented in the functions of the edgeR package by comparison iN-NGN2 samples with N-DSi samples. Genes whose transcription was altered twofold with a Benjamini-Hochberg corrected *p*-value < 0.05 were selected to evaluate hyper- and hypo-expressed genes.

Enrichment analysis of signaling and metabolic pathways (Khatri et al., 2012) was performed with the g: Profiler web service (https://biit.cs.ut.ee/gprofiler/gost) using pathway information from the KEGG database (https://www.genome.jp/kegg/pathway.html) as well as Gene Ontology biological process information (http://geneontology.org). Only pathways and processes with an adjusted p-value < 0.05 were analyzed. The default g: SCS method proposed by g: SCS was used as a correction for multiple comparisons. The treemap method implemented in the R treemap package (version 2.4–3) was used to visualize the identified pathways and processes. Signaling and metabolic pathways were grouped according to the pathway classification presented in KEGG (https://www.genome.jp/kegg/pathway.html). Gene Ontology processes were clustered using information on their position in the acyclic directed graph and hence semantic similarity of terms. Wang’s method, implemented in the calculateSimMatrix function from the rrvgo package (version 1.10.0), was used to evaluate term similarity. Process clustering was performed using the average linkage method (function hclust), and clusters were identified using the cutreeDynamic function from the dynamicTreeCut package (version 1.63–1).

## 3 Results

### 3.1 Generation and characterization of neural cultures

**Figure 1:**
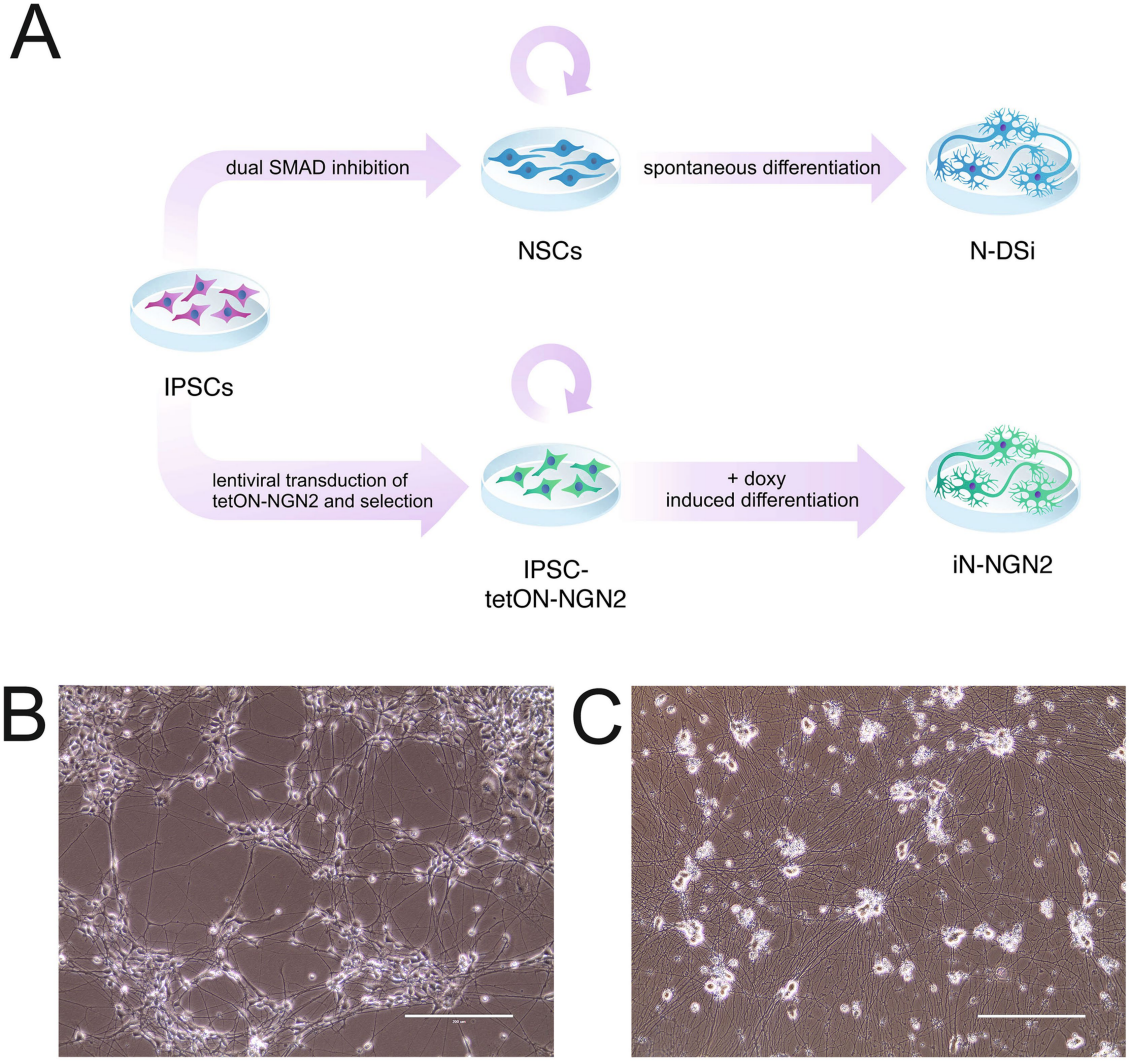
**(A)** Schematic representation for the experimental design. iPSC, induced pluripotent stem cell; NSC, neural stem cell; N-DSi, neural culture obtained from iPSC through the NSC stage using the DUAL SMAD inhibition protocol; iN-NGN2, *NGN2*-induced neural culture obtained from iPSC. **(B)** N-DSi; Phase contrast microscopy, scale bar 200 μm. **(C)** iN-NGN2; Phase contrast microscopy, scale bar 200 μm.

To obtain neural cultures using the method with DUAL SMADi (hereinafter neural cultures will be referred to as N-DSi), iPSC-KYOU were differentiated into NSCs by small molecule inhibition of two SMAD-signaling pathways. At passage 5, NSCs were directed to spontaneous differentiation (Figures 1A,B). We considered the day when NSCs were plated on medium for differentiation as day 0.

To obtain *NGN2*-induced neural cultures (hereafter referred to as iN-NGN2), we generated the transgenic line IPSC-KYOU-tetON-rtTA-NGN2. *NGN2* induction and differentiation into neurons were induced by adding doxycycline to the medium (Day 0) (Figures 1A,C).

**Figure 2:**
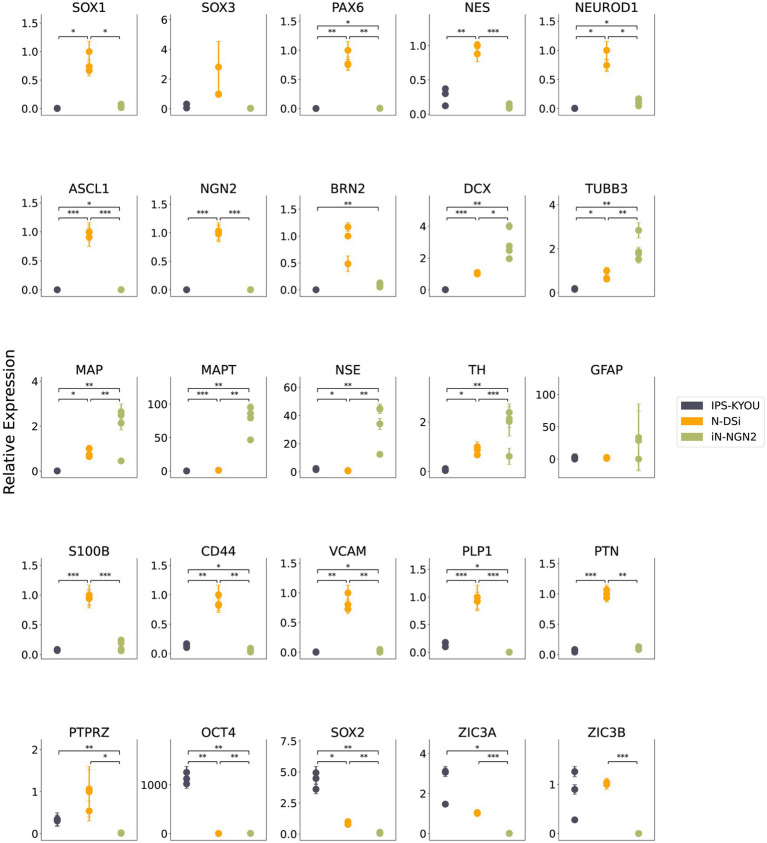
Results of quantitative RT-PCR analysis of marker gene expression levels in iPSC-KYOU (*n* = 3), N-DSi neural cultures (*n* = 3), and iN-NGN2 neural cultures (*n* = 4). The ordinate axis shows the expression level calculated by the -ΔΔCt method normalized to the expression of housekeeping genes (*EMC7* and *PSMB4*). First N-DSi sample was used as a calibrator. Each dot represents a biological replicate. Error bars indicate the standard deviation of technical replicates. Statistical significance calculated using Welch’s test is indicated by asterisks (*p-value < 0.05; **p-value < 0.01; ***p-value < 0.001).

We analyzed the relative expression levels of marker genes by quantitative RT-PCR in two-week-old neural N-DSi and iN-NGN2 neural cultures, as well as the iPSC-KYOU line, which is the common source of both neural cultures. The results showed increased expression of NSC markers (*SOX1, PAX6, NES*) and neuronal precursor markers (*NEUROD1, ASCL1, NGN2*) of N-DSi compared to iPSC and iN-NGN2 (Figure 2).

In addition, the expression of glial markers such as *S100b*, *CD44*, *VCAM1*, *PLP1*, *PTN*, and *PTPRZ* was higher in N-DSi. In contrast, neuronal markers (*TUBB3*, *MAPT*, *NSE*) as well as *DCX* (neuroblast and early neuronal marker) had higher expression levels in iN-NGN2 compared to N-DSi (Figure 2). These data indicate that non-neuronal cell types (NSCs and glial cells) predominate over neurons in N-DSi. In order to validate the heterogeneity of the studied cell cultures, and to highlight the presence of both neurons and astrocytes in N-DSi, we performed immunostaining and fluorescence microscopy (Supplementary Figure S2).

### 3.2 Transcriptome analysis of N-DSi and iN-NGN2 neural cultures

**Figure 3:**
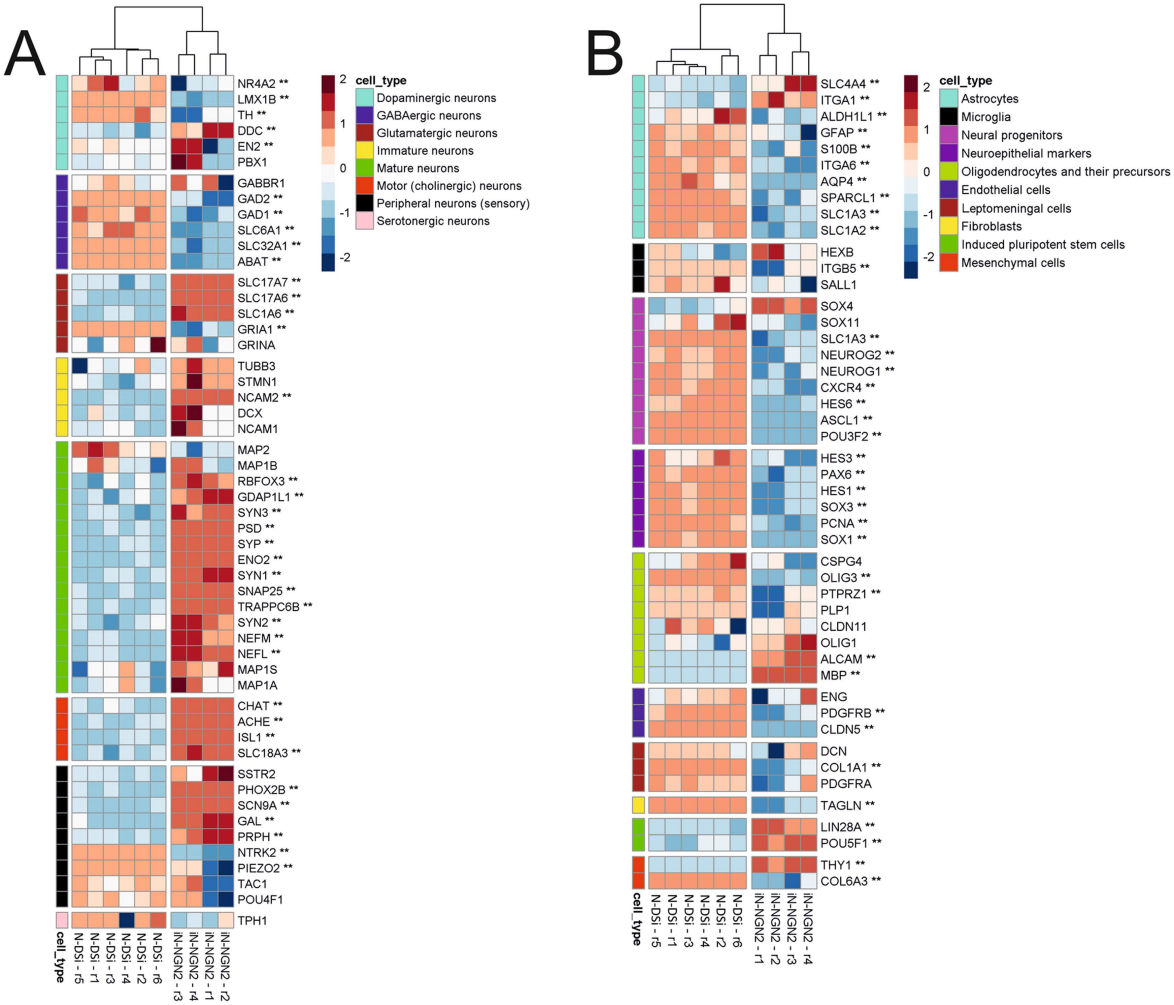
Heatmaps of change in relative expression values of manually selected cell marker genes transcription on the *z*-score scale. Two asterisks (**) indicate genes with |logFC| > 1 and BH-adjusted *p*-value < 0.05. **(A)** Heatmap of neuronal markers. **(B)** Heatmap of other cell type markers.

**Figure 4:**
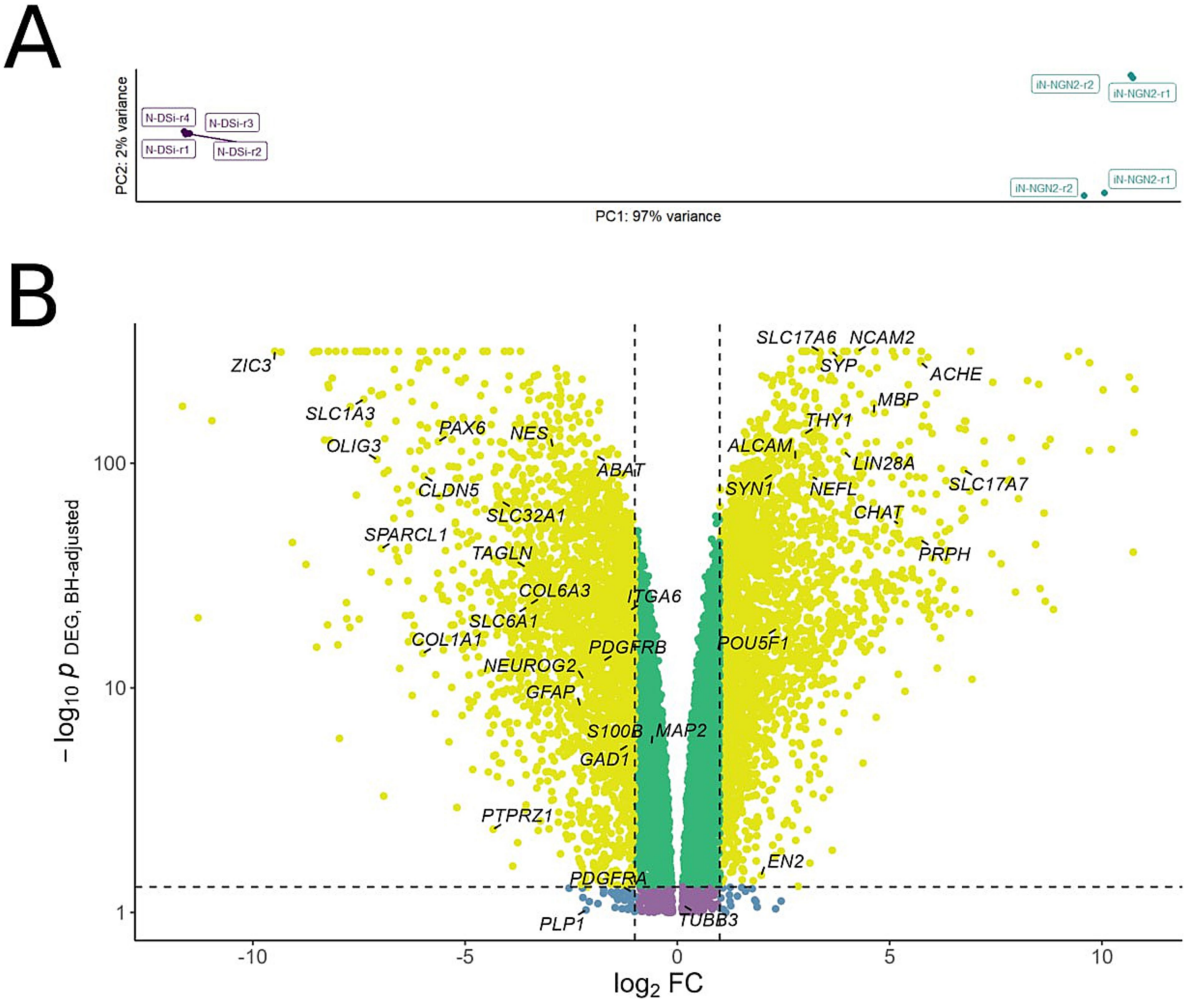
**(A)** Principal component analysis (PCA) applied to variance-stabilized transformed expression data. X and Y axes are principal components 1 and 2, respectively, explaining 97 and 2% of the variance. **(B)** Volcano plot of differentially expressed genes in iN-NGN2 compared to N-DSi. Each dot represents a gene, with coordinates indicating the effect (log_2_ fold change of expression in iN-NGN2 vs. N-DSi) vs. statistical significance (−log_10_ p-value, BH adjusted). Yellow dots correspond to genes whose expression changes both have an absolute fold change > 2 and a *p*-value < 0.05. Specific genes mentioned in the text are shown in the picture.

Transcriptome analysis included 4 samples (biological repeats) of 14-day iN-NGN2 neural cultures, and 6 samples of N-DSi neural cultures on day 14 of differentiation The results of transcriptome analysis showed that N-DSi and iN-NGN2 neural cultures differ in their transcriptome profiles (Figures 3, 4).

We evaluated the differences between N-DSi and iN-NGN2 relative gene expression of markers of different neuronal types, as well as different neural progenitors and other cell types (Figure 3; Supplementary Table S1).

iN-NGN2 differs from N-DSi by higher expression of most of the selected mature neuron marker genes such as *SYP, SYN, ENO2* (NSE), *NEFL*, et al. (Figure 3A). Moreover, the relative expression level of both mature and immature neuronal markers in iN-NGN2 is much higher than in N-DSi (Figure. 3A). Also, iN-NGN2 neural cultures are characterized by increased expression of marker genes of cholinergic and glutamatergic neurons, but not GABAergic. In addition, the expression of markers of peripheral sensory neurons (unspecified neurotransmitter specification) was detected (Figure 3A). The expression of neuroepithelial marker genes and neural progenitors of different stages is high in N-DSi compared to iN-NGN2 (Figure 3B). This suggests the presence of a significant proportion of undifferentiated neural progenitors in N-DSi neural cultures compared to iN-NGN2. In contrast to iN-NGN2, N-DSi neural cultures are significantly enriched in the expression of astrocyte markers (*S100B, GFAP, SPARCL1, SLC1A3, ITGA6,* et al.), oligodendrocytes (*PTPRZ1, PLP1, OLIG3*), and in addition, markers of other cell types such as endothelial (*PDGFRB*, *CLDN5*), vascular leptomeningeal cells and fibroblasts (*DCN*, *COL1A1*, *PDGFRA*, *TAGLN*), and stromal mesenchymal cells (*COL6A3*). This data further confirms the heterogeneity of cultures obtained through the NSC stage. In iN-NGN2, on the other hand, the expression of two iPSC markers (*LIN28A*, *POU5F1*), the mesenchymal marker *THY1* and some oligodendrocyte markers (*ALCAM, MBP*) was increased (Figure 3B).

In the PCA plot, the N-DSi and iN-NGN2 sample clusters are located at different ends of the first PC, which explains 97% of variation (Figure 4A). It can also be seen that the iN-NGN2 biological repeats are separated into 2 clusters (Figure 4A), but the corresponding second PC explains only 2% of variation. Thus, based on the obtained results, it can be stated that N-DSi and iN-NGN2 neural cultures are reliably different from each other.

Genes whose transcription was altered twofold were selected for further analysis with an adjusted *p*-value < 0.05 (Figure 4B; Supplementary Table S2). A total of 5,253 genes were identified, of which 4,974 genes (95%) are protein-coding according to Ensembl (https://www.ensembl.org/index.html).

We then identified KEGG signaling and metabolic pathways and Gene Ontology processes enriched with differentially expressed genes in iN-NGN2 neural cultures compared to N-DSi (Supplementary Figure S1). Enrichment analysis of signaling and metabolic pathways (Khatri et al., 2012) is meant to find pathways that are more enriched with differentially expressed genes under investigation compared to a reference set of genes. Neural cultures of iN-NGN2 were significantly enriched in pathways characteristic of the nervous system (pathways associated with synapses of different neurotransmitter specification, neurotrophin signaling pathway, long-term potentiation, long-term depression, etc.) and the endocrine system (estrogen, oxytocin, gonadotropin-releasing hormone (GnRH) signaling pathways, as well as insulin, GnRH, aldosterone and renin secretion) (Supplementary Figure S1A). In addition, iN-NGN2 expressed genes of pathways associated with axonal growth, various types of transduction, such as calcium transduction (Supplementary Figure S1A). At the same time, compared to N-DSi, genes related to cell cycle progression, regulation of the pluripotent state of cells, DNA replication and repair, as well as WNT and TGFβ signaling pathways are hypo-expressed here (Supplementary Figure S1B).

Similar results were observed in the top 100 Gene Ontology processes identified for hyper- and hypo-expressed genes are shown in Supplementary Figures S1C,D. Compared with N-DSi, iN-NGN2 neural cultures are enriched with processes related to intercellular interactions and signaling, synapse organizations, neuronal synaptic plasticity and transmission, various synaptic processes including exocytosis and neurotransmitter secretion, action potential generation, and axonogenesis (Supplementary Figure S1C). At the same time, processes related to cell cycle regulation and progression, mitotic processes and division, regulation of transcription, RNA and macromolecule metabolism, embryogenesis, neural tube and nervous system development, and cell adhesion and migration are more prominent in N-DSi (Supplementary Figure S1D). The detailed results of enrichment with biological processes are presented in Supplementary Tables S3, S4.

## 4 Discussion

We have compared the two most popular approaches to differentiate iPSCs into neurons: the approach with differentiation through the stage of NSCs derived from iPSCs using a method with DUAL SMAD inhibition, and the approach with induction of overexpression of exogenous *NGN2* by lentiviral delivery of a tetracycline regulated transgene expression system.

We previously found that neural cultures spontaneously differentiated from iPSC-derived NSCs using a method with DUAL SMAD inhibition are highly heterogeneous which, however, contain cells expressing key neuronal markers. Nevertheless, the composition of heterogeneous cultures was influenced by the duration of NSCs cultivation as well as the iPSC lineage, the source of NSCs (Galiakberova et al., 2023).

Neural cultures obtained from iPSCs by overexpression of exogenous *NGN2* were visually more homogeneous in cellular composition. Most of the cells of such culture had specific calcium activity and demonstrated the presence of ionotropic glutamate receptors (Galiakberova et al., 2022).

To compare the approaches, we analyzed the expression of marker genes by quantitative RT-PCR as well as the data of total transcriptome profiles of 14-day neural cultures derived from the same iPSC-KYOU line but by two different approaches. iN-NGN2, neural cultures obtained by overexpression of the *NGN2* gene in iPSC-KYOU; N-DSi, neural cultures spontaneously differentiated from NSCs (derived from iPSC-KYOU using DUAL SMADi). Although it is known that the minimum maturation period for neurons produced by DUAL SMAD inhibition is 21–28 days (Telias et al., 2014), which is almost twice as long as for neurons induced by NGN2 overexpression (14 days) (Zhang et al., 2013), a single 14-day maturation period was chosen for comparative analysis. This decision was made in order to ensure comparability of conditions, as well as to emphasize the difference in the properties of neuron cultures during the same maturation period.

The results of quantitative RT-PCR demonstrated that both neural cultures differed in the relative expression levels of neuronal markers from the iPSC-KYOU from which both neural cultures were derived. However, the level of relative expression of NSC and glial cell markers was higher in N-DSi than in iN-NGN2, and the level of neuronal markers was lower. *NGN2* expression was upregulated in N-DSi but not in iN-NGN2. This is consistent, since *NGN2* is expressed in neuronal precursors and promotes their differentiation, but is not expressed in postmitotic neurons (Hulme et al., 2022).

An interesting result is the expression of *ZIC3A* and *ZIC3B* genes in N-DSi, comparable to that in iPSCs. ZIC3A and ZIC3B are isoforms of the transcription factor ZIC3 (Bedard et al., 2011). Expression of this transcription factor may indicate cell stemness, as its activity is required during early embryonic development (gastrulation, neurulation) (Herman and El-Hodiri, 2002), and in mouse embryonic stem cells, ZIC3 maintains their pluripotency (Lim et al., 2007; Yang et al., 2019). It is also known that *ZIC3* is expressed in cells of the developing forebrain. It promotes the proliferation of neuron precursors and is also involved in their differentiation at various stages of brain development (Inoue et al., 2007; Winata et al., 2015). Thus, the expression of the isoforms of the ZIC3 gene once again emphasizes the presence of a significant proportion of proliferating stem cells in 14-day neural cultures obtained using the DUAL SMAD inhibition method.

The results of analyzing transcriptome profiles also showed significant differences between neural cultures. Metabolic and signaling pathway enrichment analysis showed that the transcriptional profile of N-DSi compared to iN-NGN2 was significantly enriched in pathways and processes related to proliferation, cell cycle maintenance, and WNT and TGFβ signaling pathways. At the same time, the transcriptional profile of iN-NGN2 was found to be enriched in various processes related to chemical synapses, neuronal plasticity, signal transduction, etc.

Compared to iN-NGN2, N-DSi cultures contained a large number of NSCs and neuronal precursors of different stages in addition to neurons. Moreover, the expression of various astroglial and oligodendrocytic genes, as well as marker genes of some other cell types, such as fibroblasts, endothelial, and vascular leptomeningeal cells, was detected. Which further confirms the heterogeneity of cultures obtained through the NSC stage.

It is generally known that NSCs derived from PSCs using a method with DUAL SMADi are capable of giving rise to both neuronal and glial branches (Chambers et al., 2009; Perriot et al., 2018; Lam et al., 2019). Based on this, various protocols have been developed to obtain astroglia from such NSCs (Perriot et al., 2018; Shimbo et al., 2020). In our experiment, we guided NSCs to spontaneous differentiation, which allowed cells to differentiate in different directions: both neuronal and glial. However, there is evidence that even when NSCs are directed to differentiate into neurons, glial cells are found in cultures (Fujimori et al., 2017; Nilsson et al., 2021). Astroglial cell markers were barely expressed in iN-NGN2, which also replicates the results of other protocols with induction of exogenous *NGN2* overexpression (Chen et al., 2020; Lin et al., 2021; Schörnig et al., 2021). On the one hand, the presence of glial cells in the culture of neurons may interfere with some studies or tests, especially if a pure culture of neurons is required. On the other hand, it is known that glial cells support neurons not only *in vivo*, but also *in vitro* (Pyka et al., 2011; Clarke and Barres, 2013). Astroglia promotes better adhesion of neurons and their survival, and also secretes various neurotrophic factors (BDNF, NGF, GDNF) that contribute to synaptogenesis and neurophysiological maturation of neurons (Pfrieger, 2010; Tang et al., 2013). In this context, the method of obtaining neurons through DUAL SMAD inhibition has an undeniable advantage.

In addition to astroglia, other cell types are found in neuron cultures obtained using the DUAL SMAD inhibition approach. One of the studies aimed at obtaining dopaminergic neurons reported that the obtained neural cultures contained not only glial but also vascular leptomeningeal cells in addition to dopaminergic neurons and their immediate precursors (Nilsson et al., 2021). In iN-NGN2, however, markers of iPSCs and mesenchymal stem cells were detected. Such non-targeted cell populations were previously detected in a study where neurons were obtained by the same approach (Lin et al., 2021). And in another study, fibroblasts were detected (Schörnig et al., 2021). In general, such collateral non-neural fibroblast-like cell types can be expected when differentiating iPSCs derived from fibroblasts by reprogramming with pluripotency factors (Buckberry et al., 2023). In neurons derived by *NGN2* overexpression, a neural stem cell population may be present as a bystander lineage (Vainorius et al., 2023), but in protocol we used the addition of AraC promotes the elimination of all actively proliferating cells, including NSCs.

Also, iN-NGN2 neural cultures, in contrast to N-DSi, demonstrated increased expression of marker genes of cholinergic and glutamatergic neurons, but expression of markers of GABAergic neurons was not observed. Interestingly, iN-NGN2 demonstrated not only cortical fate but also the fate of peripheral sensory neurons. All these results are consistent with the literature (Schuurmans et al., 2004; Nickolls et al., 2020) including those obtained by analyzing the transcriptomes of single neural culture cells derived by *NGN2* overexpression (Lin et al., 2021; Schörnig et al., 2021). In contrast, some markers of GABAergic neurons were expressed in N-DSi, suggesting that method-based protocols with DUAL SMADi are more suitable for obtaining GABAergic neurons (Liu et al., 2013; Vigont et al., 2021).

Оur study has several limitations. First, only a single iPSC line was used. While iPS-KYOU is a common iPSC line, many others are widely used, and the reported effects could differ depending on the cell background. In the future, to confirm these results, it is necessary to compare neural cultures obtained by two approaches from several different iPSC lines. Secondly, for the generation of neuronal cultures, we utilized two methods routinely used in our laboratory. For the DUAL SMAD inhibition method, we used a popular commercial kit from Thermo (Gibco) with a composition that is not publicly disclosed. Using modified protocols of similar approaches could lead to different results. Thirdly, we analyzed neural cultures with a fairly short maturation period of 14 days. Probably, when comparing neural cultures with a longer maturation stage, we also could get different results. For example, Rosa et al. obtained completely different results in the context of neuron maturity, where they compared 3-6-month-old neural cultures obtained by the DUAL SMAD inhibition approach (the protocol differed from that in this article) with 14-19-day cultures of NGN2-induced neurons (Rosa et al., 2020). Fourth, it should be noted that the set of marker genes we chose cannot fully prove the presence of certain cell types, because often a cell type is characterized not by one or two markers, but by a whole set of genes and the degree of their expression. Moreover, some of them may mark other cell types as well.

Nevertheless, based on the data obtained, it can be stated that the obtained iN-NGN2 cultures are more homogeneous compared to N-DSi. Since this study employed bulk transcriptome analysis, which aggregates data from the entire cell population, the direct assessment of neuronal maturity under the DUAL SMADi protocol was associated with a risk of insufficient sensitivity due to the potential dilution of transcripts from mature neurons. To validate the interpretation of our data, we leveraged our previously published scRNA-seq results (Galiakberova et al., 2023), obtained from an identical culture under the same conditions. It was determined that at the corresponding stage of differentiation, the proportion of mature neurons in the population did not exceed 5% (Supplementary Figure 3). This allowed us to extrapolate the findings and interpret changes in marker expression in the bulk transcriptomic data as indicators of the neuronal maturation process itself, rather than merely a consequence of shifts in cellular composition.

Thus the results of our pilot study showed that neural cultures derived from iPSCs using the DUAL SMAD inhibition method through the NSC stage (N-DSi) were more heterogeneous with neurons comprised only a small proportion (on the 14th day of maturation) compared to neural cultures obtained from iPSCs via exogenous NGN2 overexpression (iN-NGN2). Furthermore, the expression levels of mature neuronal markers were significantly higher in iN-NGN2 compared to N-DSi. Nevertheless, residual iPSC-derived cells are likely present in iN-NGN2. Although cortical excitatory (glutamatergic) neurons predominate among the neurons in the neural cultures obtained by the two different approaches, the other minor neuronal types differ between the methods. For example, the DUAL SMAD inhibition approach produces neurons of different neurotransmitter specification: GABAergic, serotonergic, and dopaminergic. In contrast, NGN2-induced neural cultures do not express markers of these types; instead, markers indicating the presence of cholinergic neurons and, in addition, peripheral sensory neurons with unspecified neurotransmitter or functional properties are detected. Based on these preliminary results, it can be concluded that, at least for the studied iPSC line (iPS-KYOU), each of the two approaches yields neural cultures with a distinct spectrum of cell types. This variability should be considered when selecting a protocol for deriving neurons from iPSCs.

## Data Availability

The datasets presented in this study can be found in online repositories. The names of the repository/repositories and accession number(s) can be found at: https://www.ncbi.nlm.nih.gov/, PRJNA846149 and https://www.ncbi.nlm.nih.gov/, PRJNA113914.
