# Supplementary material for: Transcriptomic profiling of neural cultures from the KYOU iPSC line via alternative differentiation protocols
Source: Front Mol Neurosci. 2025 Oct 28;18:1661986. doi: 10.3389/fnmol.2025.1661986 (PMC12602485; doi:10.3389/fnmol.2025.1661986)
Supplement: Supplementary file 2 [file Table_2.docx]

Supplementary Materials


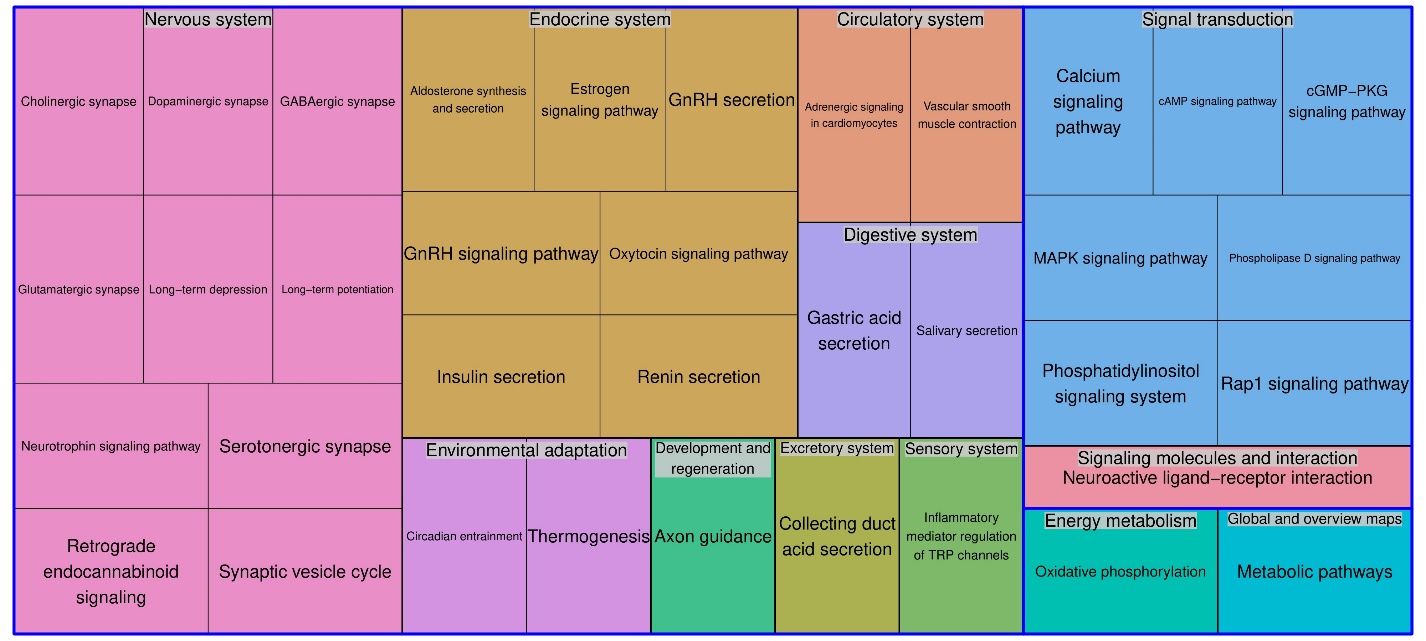


B

C


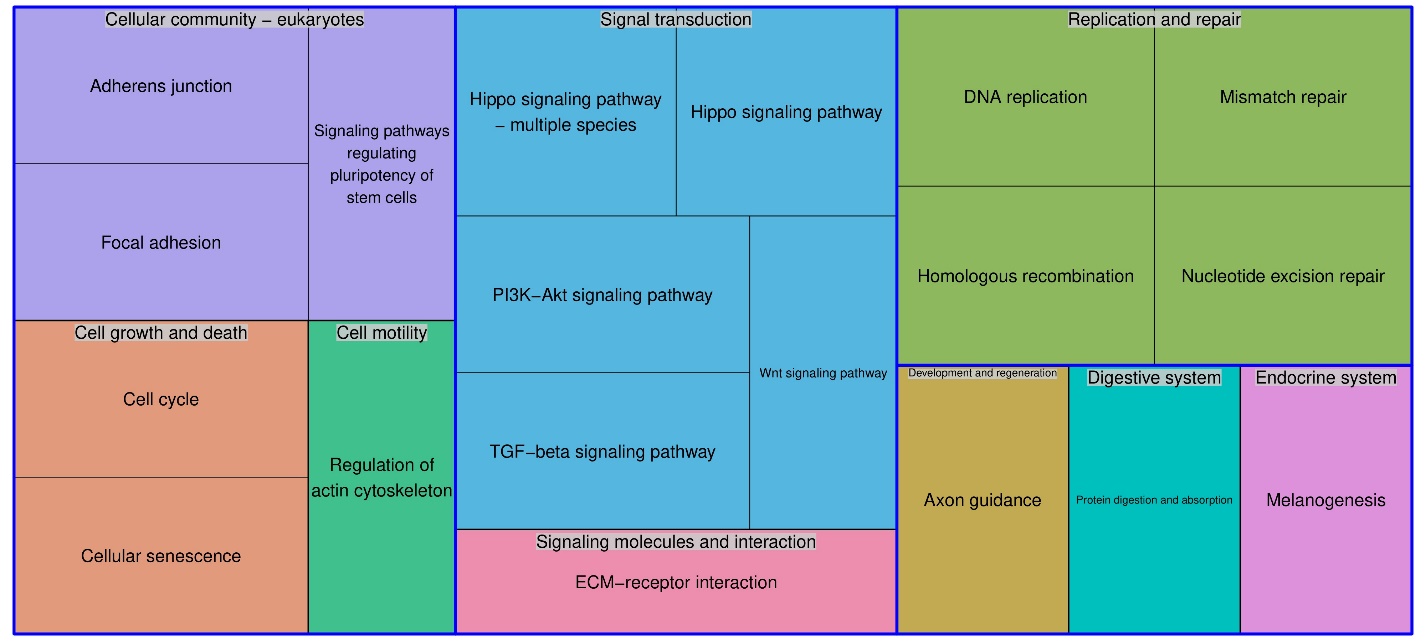


C


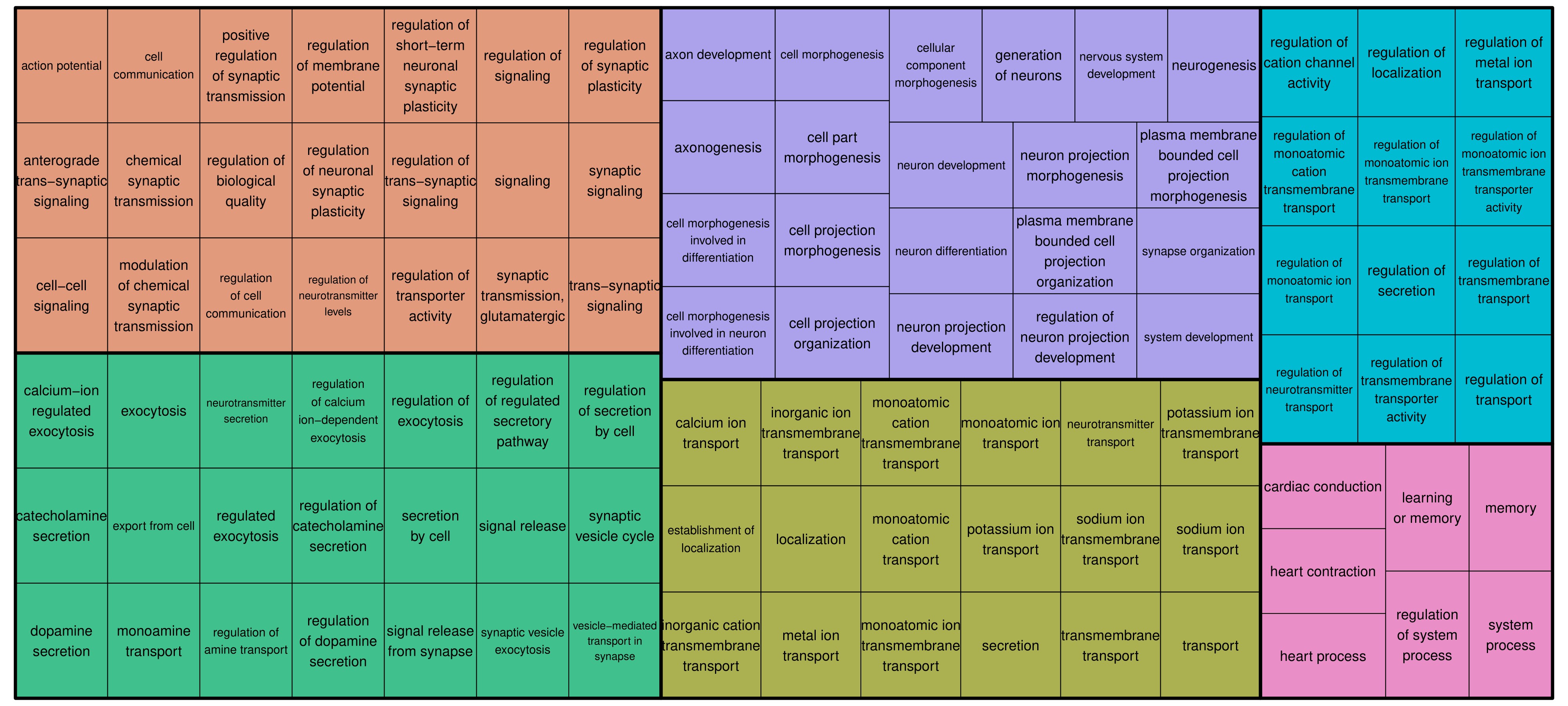


D


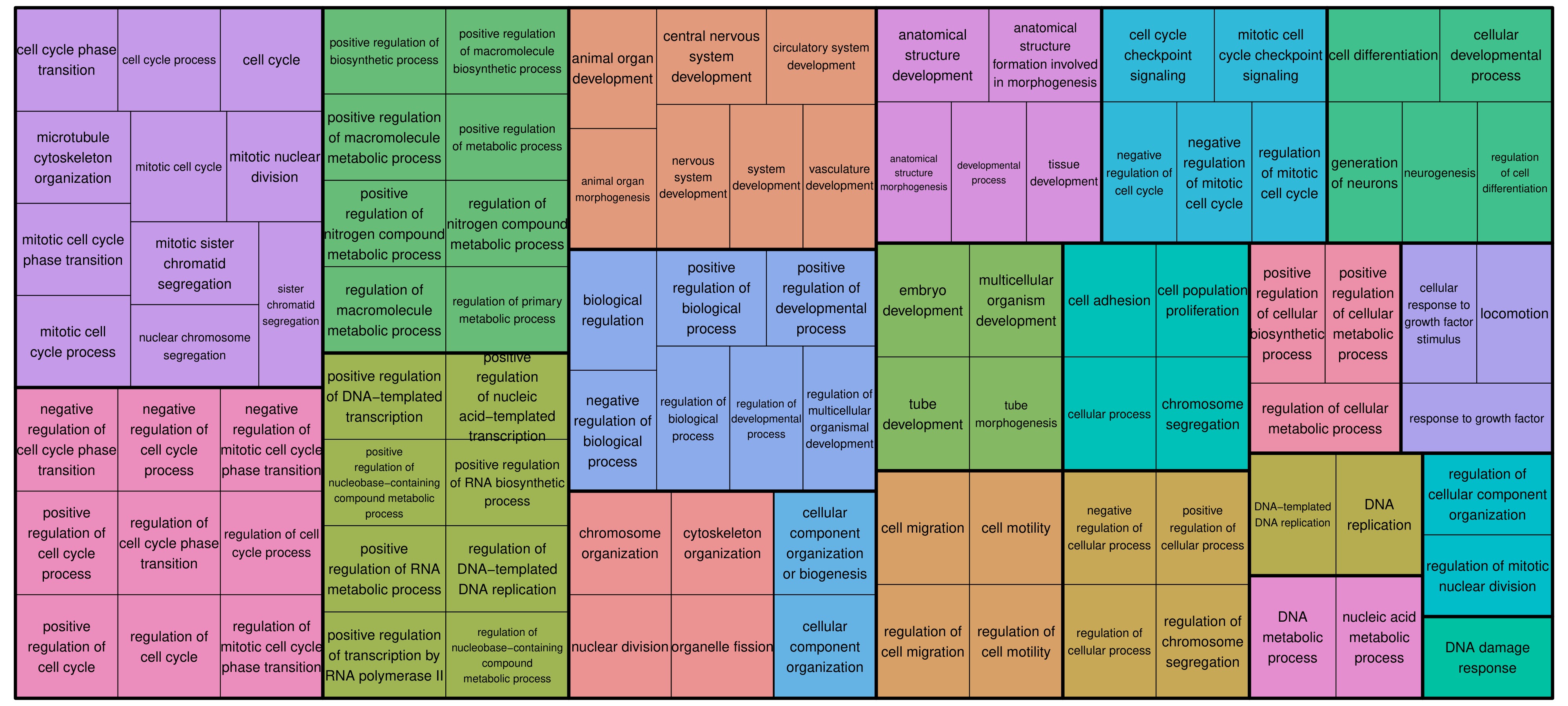


Supplementary Figure S1. Signaling and metabolic pathways KEGG and Gene Ontology Biological processes identified for differentially expressed genes in iN-NGN2 compared to N-DSi (|logFC| > 1, p < 0.05). (A) KEGG pathways for hyper-expressed genes; (B) KEGG pathways for hypo-expressed genes; (C) Gene Ontology processes for hyper-expressed genes; (D) Gene Ontology processes for hypo-expressed genes. Gene Ontology processes were clustered based on semantic similarity of terms. The complete lists of pathways and processes are presented in Supplementary Tables S3 and S4.


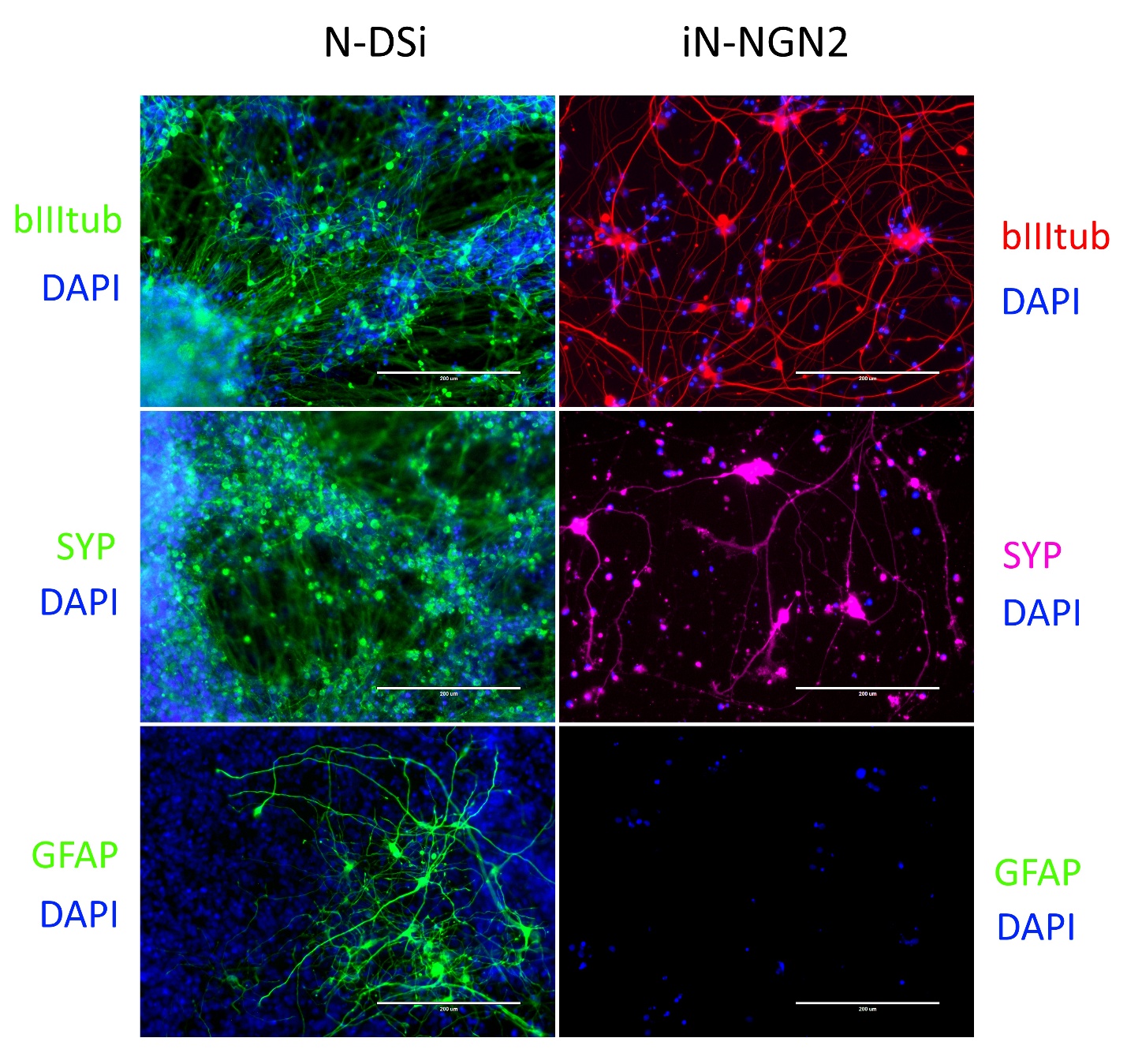


Supplementary Figure S2. Immunocytofluorescence staining of neural cultures obtained by DUAL SMAD inhibition (N-DSi) or exogenous NGN2 induction (iN-NGN2). Scale size 200µm. GFAP staining in N-DSi obtained earlier, described in the publication doi: 10.3389/fnmol.2023.1037902).


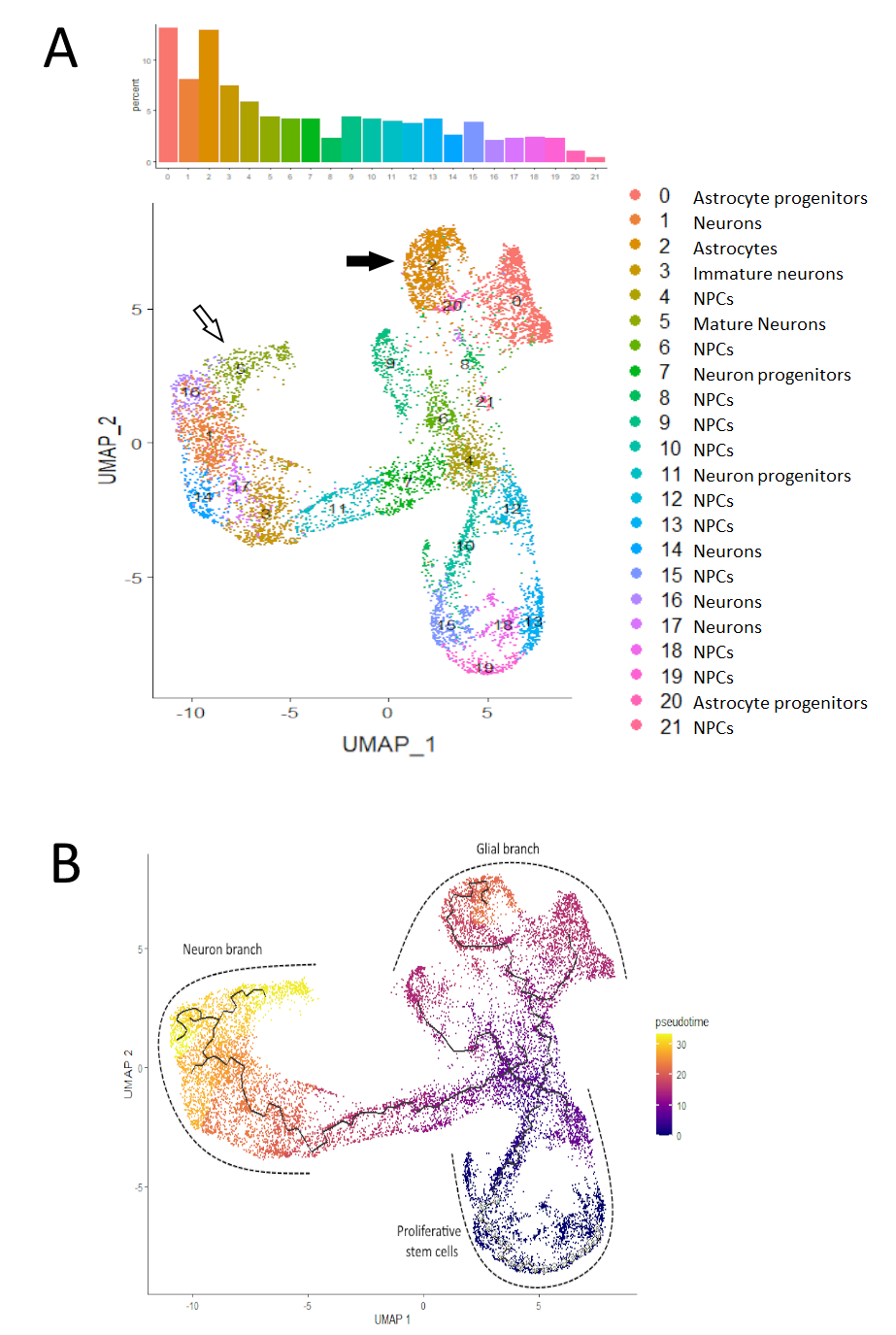


Supplementary Figure S3. Data from scRNA-seq analysis of a heterogeneous population of neural culture obtained by DUAL SMAD inhibition. (Data obtained earlier, described in the publication doi: 10.3389/fnmol.2023.1037902). А - The distribution of cells In clusters, the white arrow indicates the population of mature neurons, the black arrow indicates the population of astrocytes. B - Trajectories of neural stem cell differentiation by pseudo-time.
